# Sir William Burnett’s Patent

**Published:** 1845-07

**Authors:** 


*Sir William Burnett's Patent.*—When chemistry was making those rapid strides which astonished the world, it was looked upon as an abstract science little likely to prove of essential service for the ordinary purposes of life. What a rapid change has a few years produced; it now is the general source to which we look for the improvement of every article necessary in the various branches of art. Scientific men employ the observations which they make in the laboratory, and render them subservient to our wants and wishes. Sir William Burnett having noticed the peculiar power of chloride of zinc in rendering vegetable fibre indestructible and incorrodible, quickly invented a process by which he is enabled to preserve some of the most important materials from the ravages of dry rot, from mildew, from the effects of the insects, and even from fire. He has given us means, whose efficacy daily experience approves, of preserving ships, sails, cordage from the destruction to which they were liable. His patent has been now some time in the hands of an association; and it has been found to surpass the sanguine expectation which had been formed. The process which is now at work on a large scale, both in her Majesty's dock-yard at Portsmouth and at Poplar, is most ingenious. The hydraulic apparatus is in both places well worthy the attention of those who are interested in the public advancement, as well as those whose interests are more immediately affected by the advantages which are to be derived from the ingenuity of the discovery. The vast mass of testimony of those, who under different circumstances have tried the process, is so directly in favour of it, that no one could hesitate in giving it a fair trial. The severest tests in our naval stations have shewn that timber, canvass, cordage, and woollen cloth impregnated with the neutral salt, the basis of Sir W. Burnett's invention, become so permanently changed, that they resist the ordinary causes of decay. The preservative power diffuses itself throughout the organic structure, in consequence of mechanical means, and articles properly prepared by it cannot be eroded by fungi, or acted upon by moisture. Some of the chemists who have examined materials submitted to its action, are of opinion that a permanent chemical union takes place between the organic fibre and the metallic base of the salt; fungous vegetation, so generally destructive, seems to be completely prevented by it; exposure to the action of the atmosphere does not in any way act upon substances which have been impregnated. In the trials made in the ships in the Pacific Ocean, the result has been most satisfactory; we learn from the proper authorities, that the sails of steam vessels, although exposed to the vicissitudes of alternate wet and solar heat, have required no repairs, and that even steam and smoke have had no influence upon sail covers. Vessels employed in trade, where heat and cold alternate, ought to adopt the invention which, in China and in Africa, has proved so serviceable. It appears that salt-water increases the efficacy of the solution. One most important fact is that, unlike some of the means which have been recommended for adoption, it is perfectly innoxious, that the most delicate are not likely to have their health at all affected by it, so that it may be safely used in all steam-vessels, even where there may be collected together a large number of persons. The oxidation of metals is retarded by it, and some satisfactory experiments have shown that both copper and iron are less susceptible of rust. One of the advantages it possesses is that of rendering bilge-water less offensive. From all that we have learnt we have little doubt that it will be most extensively employed, and that the directors of railroads will be induced from the high character it has deservedly gained, to use on railways a process with which they cannot fail to be satisfied.—*Ibid, from Polytechnic Review.*

